# Advanced Glycation End Products Induce Atherosclerosis via RAGE/TLR4 Signaling Mediated-M1 Macrophage Polarization-Dependent Vascular Smooth Muscle Cell Phenotypic Conversion

**DOI:** 10.1155/2022/9763377

**Published:** 2022-01-13

**Authors:** Yujie Xing, Shuo Pan, Ling Zhu, Qianwei Cui, Zhiguo Tang, Zhongwei Liu, Fuqiang Liu

**Affiliations:** ^1^Department of Cardiology, Shaanxi Provincial People's Hospital, Xi'an, China; ^2^Cardiovascular Research Center, Shaanxi Provincial People's Hospital, Xi'an, China; ^3^Affiliated Shaanxi Provincial People's Hospital, Northwestern Polytechnical University, Xi'an, China

## Abstract

**Objective:**

The objective of this study was to investigate the involved mechanisms of advanced glycation end product- (AGE-) exacerbated atherosclerosis (AS).

**Methods:**

Toll-like receptor 4 (TLR4) inhibitor was administrated to type 2 diabetes mellitus (T2DM) AS rats. Atherosclerotic plaque, M1 macrophage infiltration, and VSMCs phenotypes were evaluated. AGE-exposed primary macrophages were treated with specific siRNAs knocking down receptor for AGEs (RAGE) and TLR4. Phenotypes of M1 macrophage and VSMCs were identified by fluorescent stains. Contact and noncontact coculture models were established. VSMCs and macrophages were cocultured in these models. ELISA was used to detect inflammatory cytokine concentrations. Relative mRNA expression levels were determined by real-time PCR. Relative protein expression and phosphorylation levels were evaluated by Western blots assays.

**Results:**

TLR4 inhibitor treatment significantly reduced arterial stenosis, infiltration of M1 polarized macrophages, and contractile-to-synthetic phenotype conversion of VSMCs in DM AS animals. *RAGE* and *TLR4* silencing dramatically reduced AGE-induced macrophage M1 polarization, inflammatory cytokine secretion, and RAGE/TLR4/forkhead box protein C2 (FOXC2)/signaling which inhibited delta-like ligand 4 (Dll4) expression in macrophages. AGE-treated macrophages induced VSMC phenotypic conversion via activating Notch pathway in a contact coculture model rather than a noncontact model. The VSMC phenotypic conversion induction capability of macrophages was attenuated by *RAGE* and *TLR4* silencing.

**Conclusions:**

AGEs induced activation of RAGE/TLR4/FOXC2 signaling, which featured macrophage with Dll4 high expression during M1 polarization. These macrophages promoted contractile-synthetic phenotypic conversion of VSMCs through the Dll4/Notch pathway after direct cell-to-cell contacts.

## 1. Introduction

Vascular smooth muscle cells (VSMCs) are located in the media of arterial vessels, maintaining the arterial structural integrity and vascular tone. A range of phenotypes have been identified in VSMCs [1]. VSMCs are quiescent and present contractile phenotype under physiological condition. When challenged by harmful stimuli, VSMCs lose contractile properties and convert into synthetic phenotype which is critical in formation and progression of atherosclerotic plaques [2]. Ours and others' previous studies suggested that delta-like ligand 4- (Dll4-) activated Notch signaling pathway was indispensable in VSMC contractile-synthetic phenotypic conversion [3–5]. It was reported that Dll4 blockage effectively attenuated several metabolic disorders including atherosclerosis [6].

Now, it is accepted that multiple immune cell types participated in the initiation and maintenance of atherosclerosis (AS) which could be recognized as the chronic inflammatory status of the arterial wall. The formation, progression, and evolution of atherosclerotic plaques are highly associated with macrophages which are differentially polarized in response to different stimuli [7]. Generally, macrophages could be divided into two polarized subgroups, namely, the classically activated (M1) and the alternatively activated (M2) macrophages. Due to their proinflammatory properties, M1 macrophages are believed to contribute to atherosclerotic lesion growth and plaque instability [8]. The quiescent macrophage (M0) to M1 polarization could be driven by various metabolites such as advanced glycation end products (AGEs) according to one of our recent investigations [9].

AGEs are sets of representative metabolites of type 2 diabetes mellitus (T2DM), which are highly associated with AS. Nonenzymatic reactions between reducing sugar and macromolecules such as nucleic acids, lipids, and amino groups take place to produce AGEs under the condition of hyperglycemia. By interacting with the receptor of AGEs (RAGE), AGEs trigger various pathological outcomes. Our recent investigation proposed that interaction between AGEs and RAGE activated toll-like receptor 4 (TLR4) signaling which facilitated macrophage M0 to M1 polarization. Our pilot study confirmed that AGE-induced M1 polarized macrophages were featured with high expression of Dll4. A previous study drew our attention, which indicated that TLR4 signaling pathway activation resulted in elevation of Dll4 expression through extracellular signal-regulated kinase (ERK)/extracellular signal-regulated kinase (FOXC2) signaling pathway [10]. Inhibition of ERK effectively suppressed plaque formation and vascular remodeling in atherosclerotic mice [11]. FOXC2 overexpression promoted atherosclerosis by facilitating cell adhesion capability [12]. Thus, it is reasonable for us to hypothesize that AGEs endow macrophages with Dll4 high expression during M0 to M1 polarization. These macrophages would further facilitate VSMCs to commit contractile-to-synthetic phenotypic conversion via activating Notch signaling.

In the present study, T2DM AS animals were administrated with TAK-242 which is the specific inhibitor of TLR4. The anti-AS effect was observed. M0 macrophages were exposed to exogenous AGEs and then coculture with primary VSMCs. The macrophage TLR4 signaling pathway was inhibited by specific small interference RNAs (siRNAs). The results from this study would add more understanding concerning the mechanisms of diabetes-associated AS and provide more clues for potential gene-targeted therapy in the future.

## 2. Materials and Methods

### 2.1. Animal Study

#### 2.1.1. Animal Model Establishment and Treatment

Six-week-old C57BL/6 background ApoE^−/−^ deficit mice purchased from Vital River (Beijing, China) were employed to establish the T2DM AS model according to previous descriptions with several modifications [13, 14]. Animals were maintained in independent polypropylene cages in controlled ambient conditions and were free to water and standard chow. Mice were weaned for 2 weeks. Intraperitoneal injections of streptozotocin (STZ, 55 mg/kg bodyweight, Sigma-Aldrich) were administrated to mice for 5 consecutive days. Then, the animals were fed Western diet (21% milk fat and 0.15% total cholesterol, TD.88137, Harlan Teklad) for 16 weeks. Control rats received intraperitoneal injections of vehicle buffer and fed Western diet. An automatic blood glucose analyzer (Roche) was used to determine fasting blood glucose (FBP) in blood sampled from the tail vein 6 weeks after STZ injections. The TLR4 inhibitor was administrated to animals according to previous descriptions with modification [15]. DM AS animals were pretreated with TAK-242 (MedChemExpress, Shanghai, China) and twice a week for 16 weeks after modeling by intraperitoneal injection (dissolved in DMSO, 3 mg/kg bodyweight) prior to STZ injections. Animal experimental protocols were reviewed and approved by Medical Research Ethics Committee of Shaanxi Provincial People's Hospital.

#### 2.1.2. Histology

The hall makers of VSMCs and macrophages were identified by immunofluorescent stains which were performed in accordance with our previous descriptions [16]. After animals were sacrificed by CO_2_ suffocation, aortic roots were dissected and fixed with paraformaldehyde (4%, *v*/*w*). 5 *μ*m paraffin tissue sections were made. For H&E stain, sections were washed by PBS and then processed with an H&E stain kit (Beyotime). The vessel lumen area (A1) and internal elastic lamina area (A2) were measured with ImageJ software (version 1.53e, NIH). The stenosis percentage was calculated as follows: (A2 − A1)/A2∗100%. Fixed cells or slides were permeabilized with 0.2% Triton X-100 (Sigma-Aldrich). After blocking buffer (Abcam) incubation, slides were washed and treated with specific primary antibodies against inducible nitric oxide synthase (iNOS, 1 : 200, Abcam), myosin heavy polypeptide 11 (MYH11, 1 : 200, Abcam), RAGE (1 : 200, Abcam), and TLR4 (1 : 200, Abcam) at 4°C for 8 h. Slides were washed by PBS and incubated with secondary antibodies conjugated to Alexa Fluor 488 (Abcam) at 25°C for 20 min. Cell nuclei were stained with DAPI (Abcam). A fluorescence microscope (Nikon) was used to observe. The captured images were further analyzed by ImageJ software.

#### 2.1.3. AGE-Bovine Serum Albumin (BSA) Preparation and Determination

The protocol of AGE-BSA preparation was adopted from our previous investigations [17, 18]. Briefly, 0.1 mmol/L glyceraldehydes (Merck) dissolved in 0.2 mmol/L NaPO_4_ buffer solution (pH = 7.4) were incubated with BSA (Hyclone) under sterile condition at 37°C for 7 d. BSA prepared without glyceraldehydes was considered as the control. The concentration of AGEs was detected by a fluorescence method with an AGEs Assay kit (BioVision). The assay is based on detecting the characteristic fluorescence (Ex/Em = 360/460 nm) shared by AGEs. A kinetic limulus amebocyte lysate (LAL) assay kit (Charles River) was used to monitor LPS in culture medium.

### 2.2. Cell Isolation and Treatment

#### 2.2.1. Mouse Primary Macrophage Isolation and Treatment

Mouse peritoneal primary macrophages (M0) were isolated. The isolation protocol was performed by following previous descriptions [19]. Briefly, 2 mL thioglycollate medium (3%, Solarbio) injections were administrated to 6-week-old C57BL/6 mice peritoneally. Three days after the thioglycollate medium injections, 3 mL peritoneal injections of 0.005% ethylenediaminetetraacetic acid were administrated. Then, thioglycollate-elicited peritoneal macrophages were harvested. After 1200g centrifugation for 5 min at 4°C, collected cells were cultured in RPMI-1640 medium (Hyclone) supplemented with 10% fetal bovine serum (FBS, Gibco) for 1 h at 37°C. The unattached cells were removed by PBS washing, and resulted cells were collected as M0 macrophages. Macrophages were identified by their surface marker F4/80 fluorescent stain. Cells were transfected with siRNAs 12 h prior to AGE-BSA exposure. Cells were incubated with AGE-BSA at concentrations of 100 and 200 *μ*g/mL for 48 h.

#### 2.2.2. Mouse Primary VSMC Isolation and Treatment

Primary mouse VSMCs were isolated by following previous descriptions [20]. Harvested whole aortas were dissected in PBS on ice. Fat and connective tissue were removed from the outside while blood and endothelial cells were scraped off from the inside. After the aortas were soaked in RPMI-1640 medium (supplemented with 1 mg/mL collagenase II and 1 U/mL elastase) for 10 min at 37°C in humidified atmosphere containing 5% CO_2_ and 95% fresh air, the adventitia was peeled off and the rest of the tissue was further treated with RPMI-1640 medium (supplemented with 2.5 mg/mL collagenase II and 2.5 U/mL elastase) for 2 h at 37°C in a humidified atmosphere containing 5% CO_2_, and 95% fresh air till single cell suspension was reached. Dissociated VSMCs were cultured in RPMI-1640 medium supplemented with 10% FBS, 2 mmol/L glutamine (Sigma-Aldrich), and antibiotics mix (Sigma-Aldrich) at 37°C in a humidified atmosphere containing 5% CO_2_ and 95% fresh air. The VSMCs were identified by their surface marker MHY11 fluorescent stain.

### 2.3. Coculture Models

**Figure 1:**
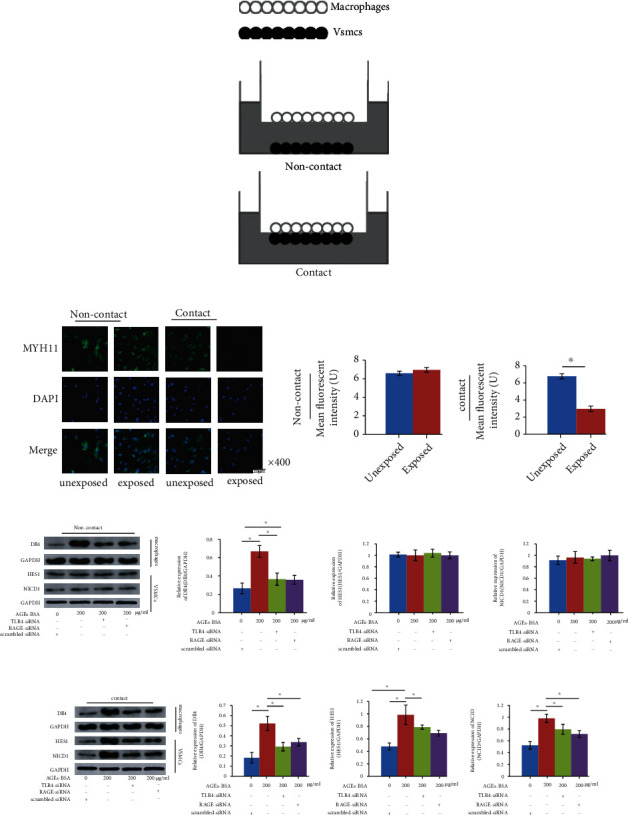
(a) Schematic diagram of non-contact and contact coculture models. (b) Captured immunofluorescent staining images of MYH11 which is the hall marker of vascular smooth muscle cells (VSMCs) of contractile phenotype. DAPI and their merged images were also demonstrated. Columns on the right panel indicate the mean fluorescent intensities of MYH11 in contact and noncontact coculture models, respectively. (c) Immunoblots of Dll4, GAPDH in macrophages and HES1, NICD1, and GAPDH in VSMCs in the noncontact coculture model. Columns indicate the relative expression levels of Dll4 in macrophages, HES1, and NICD1 in VSMCs in the noncontact coculture model. (d) immunoblots of Dll4, GAPDH in macrophages and HES1, NICD1, and GAPDH in VSMCs in the contact coculture model. Columns indicate the relative expression levels of Dll4 in macrophages, HES1, and NICD1 in VSMCs in the contact coculture model. *n* = 6; ^∗^*P* < 0.05.

Contact and noncontact coculture models were adopted from our previous investigation [5] which was demonstrated in Figure 1(a). According to previous descriptions, macrophages were cocultured with VSMCs in Millicell-24 Cell Culture Insert Plate system with polyethylene terephthalate membranes (Millipore) [21, 22]. For the contact coculture model, macrophages (4 × 10^4^/cm^2^) were seeded to the basal surface of the insert. Then, the insert was reverted into 6-well culture plates. VSMCs (4 × 10^4^/cm^2^) were seeded to the apical surface of the insert. Then, the system was incubated in RPMI-1640 medium supplemented with 10% FBS, 2 mmol/L glutamine, and antibiotics mix for 72 h at 37°C. For the noncontact model, macrophages (4 × 10^4^/cm^2^) were seeded to the bottom of the well.

### 2.4. siRNA Transfections

Expressions of RAGE and TLR4 in macrophages were knocked down by RNA interfering (RNAi) technique. siRNAs were designed and synthesized by TaKaRa (Japan). The targeting sequence of siRNA for RAGE was 5′-GGAATGGAAAGGAGACCA-3′ [23] and for TLR4 was 5′-CGGCGAAGTAAAGAATCTGAA-3′ [9]. Scrambled siRNA (Thermo) was used as the negative control. These siRNAs were transfected into macrophages with HiPerFect siRNA transfection reagent (Qiagen) according to the manufacturer's instructions.

### 2.5. Real-Time Polymerase Chain Reaction (RT-PCR)

RT-PCR was carried out to determine the expression levels of *RAGE*, *TLR4*, *FOXC2*, and *Dll4* in macrophages, respectively. *GAPDH* was used as the internal reference. Total RNA was extracted by using TRIzol reagent (TaKaRa). cDNA transcription was performed with PrimeScript RT Master Mix (TaKaRa). RT-PCR was performed by using SYBR Premix Ex Taq™ II (TaKaRa). Gene expression levels were calculated by 2^-△△ct^ method and presented as fold changes. Primers were synthesized by TaKaRa and listed as below. The primer sequence for *RAGE* was as follows: forward—5′-GGACCCTTAGCTGGCACTTAGA-3′; reverse—5′-GAGTCCCGTCTCAGGGTGTCT-3′. The primer sequence for *TLR4* was as follows: forward—5′-AGACATCCAAAGGAATACTGCAA-3′; reverse—5′-GCCTTCATGTCTATAGGTGATGC-3′. The primer sequence for *GAPDH* was as follows: forward—5′-CTCCATTCCTCCTCCAGACACT-3′; reverse—5′-GCCTTCATGTCTATAGGTGATGC-3′.

### 2.6. Western Blots

Cells were rinsed with Dulbecco's PBS and lysed by using RIPA lysis buffer system with PMSF (Santa Cruz) on ice. Proteins were extracted by using a Total Protein Extraction Kit (Beyotime). Concentrations of protein samples were detected by using a BCA kit (Invitrogen). Proteins were separated by 10% SDS-PAGE and then transferred to PVDF membranes. After blocking buffer (Abcam) incubation for 1 h, membranes were further incubated with specific primary antibodies against RAGE (1 : 500, Abcam), TLR4 (1 : 500, Abcam), phosphorylated ERK (p-ERK, 1 : 500, Abcam), ERK (1 : 500, Abcam), FOXC2 (1 : 1000, Abcam), Dll4 (1 : 1000, Abcam), NICD1 (1 : 1000, Abcam), HES1 (1 : 1000, Abcam), and GAPDH (1 : 2000, Abcam) at 4°C for 8 h. After TBST washing, the membranes were incubated with secondary antibodies at 25°C for 1 h. Western Blotting Luminal Reagent (Santa Cruz) was used to develop the membranes, and the immunoblots were visualized on X-ray films. The optic densities of the blots were analyzed by using ImageJ Software.

### 2.7. Enzyme-Linked Immunosorbent Assay (ELISA)

Concentrations of interleukin (IL)1*β*, IL6, and tumor necrosis factor (TNF)*α* in cell culture medium supernatant were detected by commercially available ELSIA kits, namely, mouse IL1*β* ELISA kit (Solarbio, detection limit: 2.5-160 pg/mL), mouse IL6 ELISA kit (Abcam, detection limit: 15.6-1000 pg/mL), and mouse TNF*α* ELISA kit (Beyotime, detection limit 26-500 pg/mL). The detection procedures were carried out according to the protocol provided by the manufacturers.

### 2.8. Statistics

Data were presented in a (mean ± SD) manner and analyzed by SPSS software (version 17.0). Student's *t*-test and one-way analysis of variance (ANOVA) were used to analyze the difference between two and multiple groups. Bonferroni's test was employed as post hoc test. Differences were statistically significant when *P* < 0.05.

## 3. Results

### 3.1. TLR4 Inhibition Suppressed Arterial Stenosis, M1 Macrophage Infiltration, and VSMC Phenotypic Conversion in Arterial Atherosclerotic Lesions in AS DM Animals Which Were Characterized with High Serum AGE Concentration

**Figure 2:**
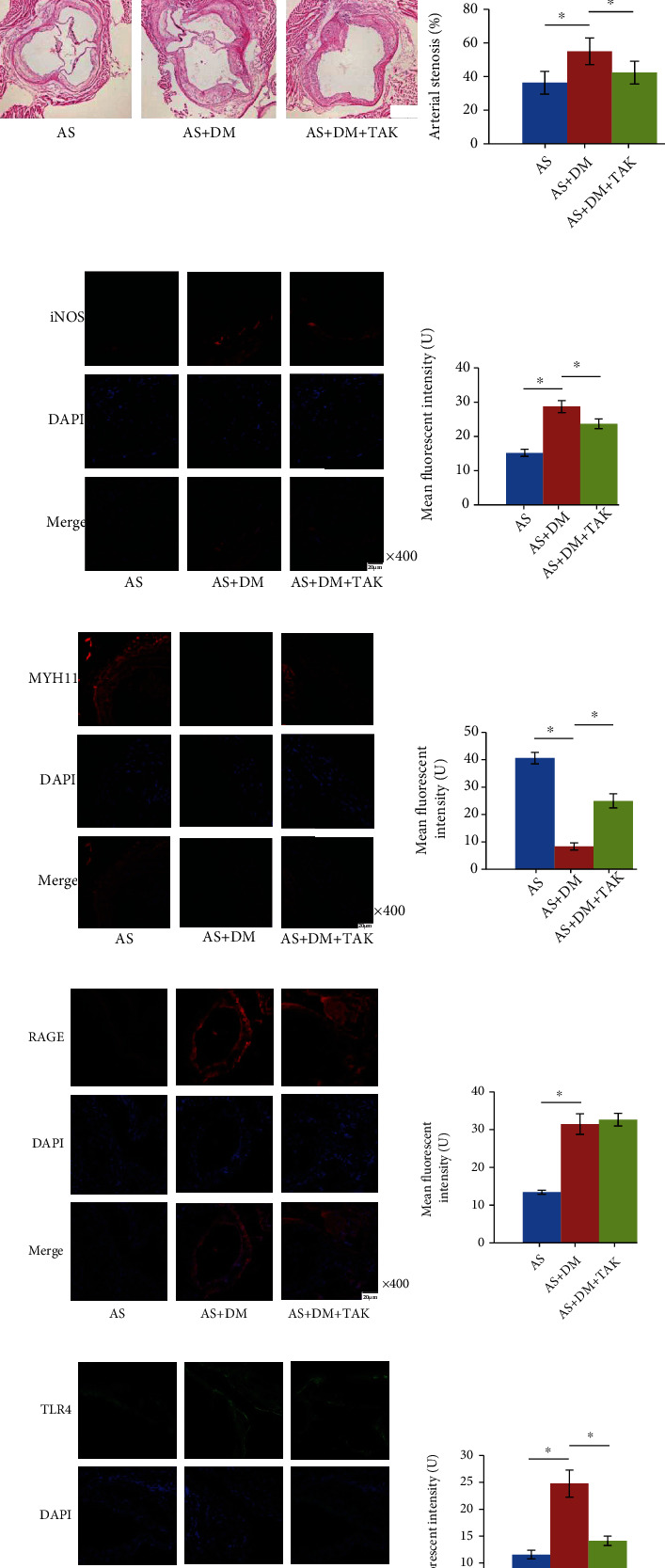
(a) Columns indicated fasting blood glucose (FGB) and serum AGE concentrations. (b) H&E staining of aortic root showing the atherosclerotic plaques. Columns indicate the calculated arterial stenosis caused by atherosclerosis. (c) Captured immunofluorescent staining images of iNOS which is the marker of M1 polarized macrophages. DAPI and their merged images were also demonstrated. Columns on the right panel indicate the mean fluorescent intensities of iNOS. (d) Captured immunofluorescent staining images of MYH11 which is the marker of vascular smooth muscle cells (VSMCs) of contractile phenotype. DAPI and their merged images were also demonstrated. Columns on the right side indicate the mean fluorescent intensities of MYH11. (e) Captured immunofluorescent staining images of RAGE. DAPI and their merged images were also demonstrated. Columns on the right side indicate the mean fluorescent intensities of RAGE. (f) Captured immunofluorescent staining images of TLR4. DAPI and their merged images were also demonstrated. Columns on the right side indicate the mean fluorescent intensities of TLR4. AS: atherosclerotic mice; AS+DM: atherosclerotic diabetic mice; AS+DM+TAK: atherosclerotic diabetic mice treated with TAK-242. *n* = 6; ^∗^*P* < 0.05.

Dramatically increased serum fasting blood glucose (FBG) and AGE concentrations were found in AS DM mice compared with AS control (Figure 2(a)). Significantly increased arterial stenosis, iNOS, RAGE, TLR4 expressions, and decreased MYH11 expression were found in aortic roots sampled from AS DM animals (Figures 2(b)–2(d)). The administration of TLR4 inhibitor TAK-242 failed to affect serum FBG and AGE concentrations in AS DM mice (Figure 2(a)). Significantly reduced arterial stenosis, iNOS, and TLR4 expressions but increased MYH11 expression were found in AS lesions in TAK-242-administrated AS DM mice (Figures 2(b)–2(d)).

### 3.2. Targeted Silencing of RAGE/TLR4 Pathway Suppressed AGE-BSA-Induced Macrophage M0 to M1 Polarization

**Figure 3:**
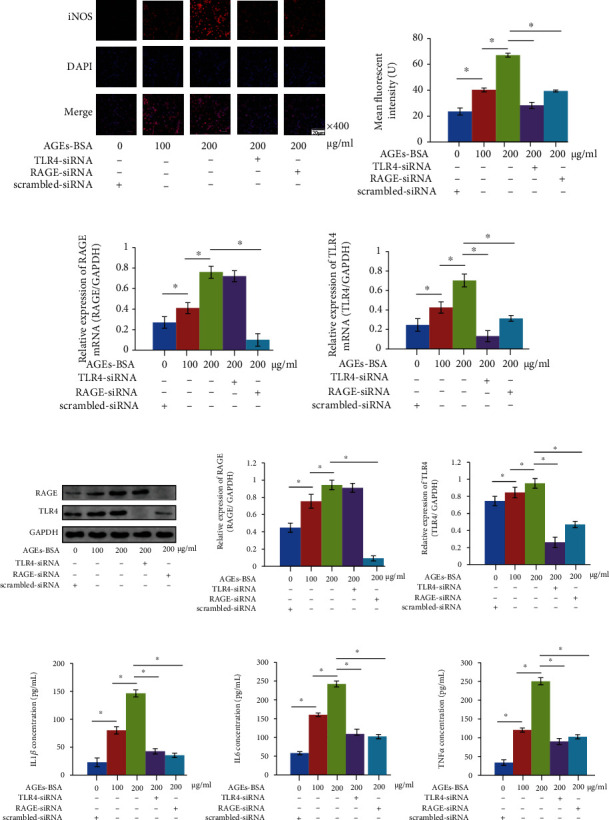
(a) Captured immunofluorescent staining images of iNOS which is the marker of M1 macrophage. DAPI and their merged images were also demonstrated. Columns on the right panel indicate the mean fluorescent intensities of iNOS. (b) Columns indicated the relative mRNA expression levels of *RAGE* and *TLR4* in macrophages. (c) Immunoblots of RAGE, TLR4, and GAPDH in macrophages were demonstrated. Columns on the right panel indicate the relative expression levels of RAGE and TLR4 in macrophages. (d) Columns indicate the concentrations of IL1*β*, IL6, and TNF*α* in cell culture medium supernatant of macrophages. *n* = 6; ^∗^*P* < 0.05.

Primary M0 macrophages were exposed to AGE-BSA at 0, 100, and 200 *μ*g/mL for 48 h. As demonstrated in Figure 3(a), the AGE-BSA exposure increased the expression of iNOS which is believed as the marker of macrophage M1 phenotype in an AGE-BSA concentration-dependent manner. Meanwhile, as demonstrated in Figures 3(b) and 3(c), AGE-BSA exposure increased expression levels of RAGE and TLR4 in macrophages in an AGE-BSA concentration-dependent manner. The exposure also increased the secretion of proinflammatory cytokines including IL1*β*, IL6, and TNF*α* in an AGE-BSA concentration-dependent manner (Figure 3(d)). Targeted silencing of RAGE and TLR4 was proved effective (Figures 3(b) and 3(c)) in decreasing iNOS expression (Figure 3(a)) and proinflammatory cytokine concentration (Figure 3(d)) in macrophages exposed to AGE-BSA at 200 *μ*g/mL.

### 3.3. RAGE/TLR4 Pathway Silencing Reduced Dll4 Expression in AGE-BSA-Incubated Macrophages

**Figure 4:**
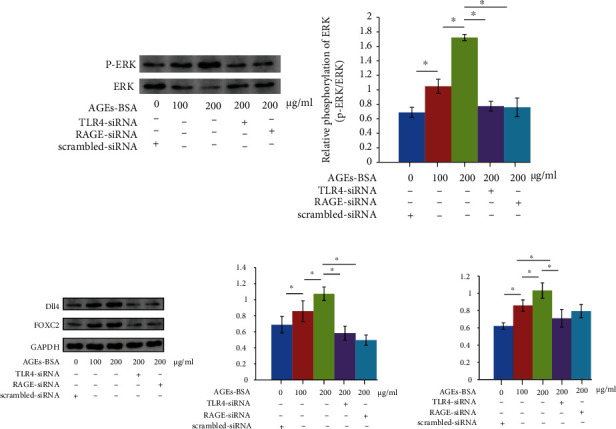
(a) Immunoblots of ERK and p-ERK were demonstrated on the left panel. Columns indicate the relative phosphorylation levels ERK in macrophages. (b) Immunoblots of FOXC2, Dll4, and GAPDH. Columns on the right panel indicate the relative expression levels of FOXC2 and Dll4 in macrophages. *n* = 6; ^∗^*P* < 0.05.

AGE-BSA exposure significantly increased ERK phosphorylation levels (Figure 4(a)) as well as FOXC2 and Dll4 expression levels (Figure 4(b)) in macrophages in an AGE-BSA concentration-dependent manner. RAGE and TLR4 silencing dramatically inhibited ERK phosphorylation (Figure 4(a)) and reduced expression levels of FOXC2 and Dll4 (Figure 4(b)) in macrophages exposed to AGE-BSA at 200 *μ*g/mL.

### 3.4. Silencing of RAGE/TLR4 Pathway Impaired the Capability of AGE-Exposed Macrophages in Inducing VSMC Contractile-Synthetic Phenotypic Conversion in Contact Coculture Model

The established contact and noncontact coculture models are demonstrated in Figure 1(a). As shown in Figure 1(b), in the noncontact model, both AGE-BSA-exposed and AGE-BSA-unexposed macrophages failed to alter MYH11 expressions in VSMCs. However, in the contact model, AGE-BSA-exposed macrophage significantly induced VSMCs to committee phenotypic conversion which was evidenced by alterations of MYH11 expression in VSMCs. As shown in Figure 1(c), in the contact coculture model, VSMCs cocultured with *RAGE*- and *TLR4*-silenced AGE-BSA-exposed macrophages presented decreased expression levels of NICD1 and HES which were the markers of activation of Notch signaling pathway. In the noncontact model, however, NICD1 and HES1 expression levels were unaltered in VSMCs (Figure 1(d)).

## 4. Discussion

AGEs are characterized pathological toxic metabolites of DM and highly associated with the adverse cardiovascular events caused by AS [17, 24]. Evidence from our previous study proved that AGEs induced macrophages M0 to M1 polarization via the RAGE/TLR4 signaling pathway [9]. M1 macrophages are predominant phenotype, playing critical roles in the occurrence and development of atherosclerotic plaques. This subgroup of macrophages infiltrates and accumulates in atherosclerotic plaques, contributing to AS-related events such as necrotic core formation, plaque rupture, and coagulation cascade activation [25, 26]. Atherosclerotic plaque M1 macrophages are characterized its specific marker iNOS [27]. In the present study, established AS DM mouse models were featured with obviously elevated serum AGE concentration and atherosclerotic plaque-caused arterial stenosis. The specific TLR4 inhibitor TAK-242 was administrated to AS DM mice to suppress the activation of TLR4 signaling. We found that TAK-242 treatment effectively reduced plaque M1 macrophage infiltration and arterial stenosis. More importantly, TLR4 inhibition attenuated MYH11 expression reduction in atherosclerotic plaques, suggesting that TLR4 inhibitor suppressed VSMC contractile-phenotypic conversion which is critical in plaque formation and progression.

Belonging to the forkhead family of transcription factors and characterized by a DNA-binding forkhead domain, FOXC2 is a key factor in vascular development and integrity [28]. Previous reports proposed that Dll4 expression was dependent on FOXC2 activation which was regulated by ERK in mice [29]. A chromatin immunoprecipitation (ChIP) study suggested that FOXC2 promoted Dll4 expression by binding to *Dll4* promoter [10]. Activation of TLR4 pathway could lead to activation of multiple nuclear factors, such as NF-*κ*B, MPAKs, and ERK [30, 31]. Similar with our previous investigation [9], in the present study, AGE-BSA exposure was used to induce the activation of RAGE/TLR4 pathway in macrophages. We found that ERK phosphorylation was facilitated as a consequence, which leads to increased FOXC2 and Dll4 expressions in macrophages. Moreover, our data showed that RAGE/TLR4 silencing by specific siRNAs resulted in the suppression of macrophage M1 polarization. Meanwhile, the activation of ERK/FOXC2/Dll4 pathway was inhibited. These results suggested that AGE-BSA stimulation featured M1 macrophages with high Dll4 expression via activating RAGE/TLR4 pathway.

VSMCs play important roles in AS. According to ours and others' previous studies, the contractile-synthetic phenotypic conversion was critical in atherosclerotic plaque formation and development [3, 32]. Synthetic VSMCs are capable of producing inflammatory cytokines and extracellular matrix which are fundamental for AS. Previous studies including ours suggested that Dll4/Notch signaling activation was highly involved in VSMC contractile-synthetic phenotypic conversion [3, 33, 34]. In the present study, AGE-BSA-exposed macrophages were characterized with Dll4 high expression. When they were cocultured with VSMCs in a contact coculture model, phenotypic conversion of VSMCs was induced. In the same contact coculture model, however, *RAGE* and *TLR4* silenced AGE-BSA-exposed macrophages failed to induce VSMC phenotypic conversion due to lose of Dll4 high-expression phenotype. These results suggested that AGE-BSA stimulation featured M1 macrophages with Dll4 high expression which further induced VSMC phenotypic conversion via direct cell-to-cell contact by activating Notch pathway.

In summary, our data suggested a novel molecular mechanism interpreting how DM exacerbates AS. AGEs fostered by DM induce activation of RAGE/TLR4 pathway in plaque macrophages which perform M1 polarization. During this process, activated RAGE/TLR4 signaling promotes Dll4 expression via the ERK/FOXC2 pathway in this subgroup of plaque M1 macrophages. By direct cell-to-cell contact, Dll4 expressed on macrophage medicated VSMCs to accomplish contractile-phenotypic conversion via the Notch pathway, contributing to AS. TLR4 signaling inhibition makes M1 macrophage lose the Dll4 high-expression phenotype. Thus, the VSMC phenotypic conversion is suppressed, and diabetes-exacerbated AS is attenuated.

## 5. Limitation

There are several limitations of this study. First, results from the in vivo study were not solid enough to support the conclusion. It would be better if TLR4 knockout or knockin animals could be used. Second, as the markers of VSMCs are changing due to phenotypic conversion. Thus, there currently are no ideal housekeeping markers for VSMCs. It would be better to insert a reporter gene (i.e., GFP) and lineage tracing reporter animal to trace all VSMCs *in vitro* and *in vivo*.

## Data Availability

Data would be available upon reasonable requests.

## Additional Points

*Animal Studies*. Animals were purchased from Vitalriver (Beijing, China). The research protocols concerning animal studies were reviewed and approved by Institutional Animal Care and Use Committee of Shaanxi Provincial People's Hospital.
